# First asteroid gas sample delivered by the Hayabusa2 mission: A treasure box from Ryugu

**DOI:** 10.1126/sciadv.abo7239

**Published:** 2022-11-16

**Authors:** Ryuji Okazaki, Yayoi N. Miura, Yoshinori Takano, Hirotaka Sawada, Kanako Sakamoto, Toru Yada, Keita Yamada, Shinsuke Kawagucci, Yohei Matsui, Ko Hashizume, Akizumi Ishida, Michael W. Broadley, Bernard Marty, David Byrne, Evelyn Füri, Alex Meshik, Olga Pravdivtseva, Henner Busemann, My E.I. Riebe, Jamie Gilmour, Jisun Park, Ken-ichi Bajo, Kevin Righter, Saburo Sakai, Shun Sekimoto, Fumio Kitajima, Sarah A. Crowther, Naoyoshi Iwata, Naoki Shirai, Mitsuru Ebihara, Reika Yokochi, Kunihiko Nishiizumi, Keisuke Nagao, Jong Ik Lee, Patricia Clay, Akihiro Kano, Marc W. Caffee, Ryu Uemura, Makoto Inagaki, Daniela Krietsch, Colin Maden, Mizuki Yamamoto, Lydia Fawcett, Thomas Lawton, Tomoki Nakamura, Hiroshi Naraoka, Takaaki Noguchi, Hikaru Yabuta, Hisayoshi Yurimoto, Yuichi Tsuda, Sei-ichiro Watanabe, Masanao Abe, Masahiko Arakawa, Atsushi Fujii, Masahiko Hayakawa, Naoyuki Hirata, Naru Hirata, Rie Honda, Chikatoshi Honda, Satoshi Hosoda, Yu-ichi Iijima, Hitoshi Ikeda, Masateru Ishiguro, Yoshiaki Ishihara, Takahiro Iwata, Kosuke Kawahara, Shota Kikuchi, Kohei Kitazato, Koji Matsumoto, Moe Matsuoka, Tatsuhiro Michikami, Yuya Mimasu, Akira Miura, Tomokatsu Morota, Satoru Nakazawa, Noriyuki Namiki, Hirotomo Noda, Rina Noguchi, Naoko Ogawa, Kazunori Ogawa, Tatsuaki Okada, Chisato Okamoto, Go Ono, Masanobu Ozaki, Takanao Saiki, Naoya Sakatani, Hiroki Senshu, Yuri Shimaki, Kei Shirai, Seiji Sugita, Yuto Takei, Hiroshi Takeuchi, Satoshi Tanaka, Eri Tatsumi, Fuyuto Terui, Ryudo Tsukizaki, Koji Wada, Manabu Yamada, Tetsuya Yamada, Yukio Yamamoto, Hajime Yano, Yasuhiro Yokota, Keisuke Yoshihara, Makoto Yoshikawa, Kent Yoshikawa, Shizuho Furuya, Kentaro Hatakeda, Tasuku Hayashi, Yuya Hitomi, Kazuya Kumagai, Akiko Miyazaki, Aiko Nakato, Masahiro Nishimura, Hiromichi Soejima, Ayako Iwamae, Daiki Yamamoto, Kasumi Yogata, Miwa Yoshitake, Ryota Fukai, Tomohiro Usui, Trevor Ireland, Harold C. Connolly, Dante S. Lauretta, Shogo Tachibana

**Affiliations:** ^1^ Department of Earth and Planetary Sciences, Kyushu University, Fukuoka 819-0395, Japan.; ^2^ Earthquake Research Institute, The University of Tokyo, Tokyo 113-0032, Japan.; ^3^ Biogeochemistry Research Center, Japan Agency for Marine-Earth Science and Technology (JAMSTEC), Yokosuka, Kanagawa 237-0061, Japan.; ^4^ Institute of Space and Astronautical Science, Japan Aerospace Exploration Agency (JAXA), Sagamihara 252-5210, Japan.; ^5^ Department of Chemical Science and Engineering, Tokyo Institute of Technology, Yokohama, Kanagawa 226-8503, Japan.; ^6^ Research Institute for Global Change, JAMSTEC, Yokosuka 237-0061, Japan.; ^7^ Institute for Extra-cutting-edge Science and Technology Avant-garde Research (X-star), JAMSTEC, Yokosuka 237-0061, Japan.; ^8^ Faculty of Science, Ibaraki University, Mito 310-8512, Japan.; ^9^ Department of Earth Science, Tohoku University, Sendai 980-8578, Japan.; ^10^ Université de Lorraine, CNRS, CRPG, F-54000 Nancy, France.; ^11^ Physics Department, Washington University, St. Louis, MO 63130, USA.; ^12^ Institute of Geochemistry and Petrology, Eidgenössische Technische Hochschule (ETH) Zürich, 8092 Zürich, Switzerland.; ^13^ Department of Earth and Environmental Sciences, The University of Manchester, Manchester M13 9PL, UK.; ^14^ Department of Physical Sciences, Kingsborough Community College, The City University of New York, Brooklyn, NY 11235, USA.; ^15^ Department of Earth and Planetary Sciences, American Museum of Natural History, NY 10024, USA.; ^16^ Department of Earth and Planetary Sciences, Hokkaido University, Sapporo 060-0810, Japan.; ^17^ Astromaterials Research and Exploration Science, Mail Code XI2, National Aeronautics and Space Administration (NASA) Johnson Space Center, Houston, TX 77058, USA.; ^18^ Institute for Integrated Radiation and Nuclear Science, Kyoto University, Osaka 590-0494, Japan.; ^19^ Faculty of Science, Yamagata University, Yamagata 990-8560, Japan.; ^20^ Graduate School of Science and Engineering, Tokyo Metropolitan University, Hachioji, Tokyo 192-0397, Japan.; ^21^ Department of Chemistry, Faculty of Science, Kanagawa University, Hiratsuka, Kanagawa 259-1293, Japan.; ^22^ Department of the Geophysical Sciences, The University of Chicago, Chicago, IL 60637, USA.; ^23^ Space Sciences Laboratory, University of California, Berkeley, CA 94720, USA.; ^24^ Division of Earth Sciences, Korea Polar Research Institute, Incheon 21990, Korea.; ^25^ School of Science, The University of Tokyo, Tokyo 113-0033, Japan.; ^26^ Department of Physics and Astronomy, Purdue University, West Lafayette, IN 47907, USA.; ^27^ Department of Earth, Atmospheric, and Planetary Sciences, Purdue University, West Lafayette, IN 47907, USA.; ^28^ Department of Earth and Environmental Sciences, Nagoya University, Nagoya 464-8601, Japan.; ^29^ Division of Earth and Planetary Sciences, Kyoto University, Kyoto 606-8502, Japan.; ^30^ Department of Earth and Planetary Systems Science, Hiroshima University, Higashi-Hiroshima 739-8526, Japan.; ^31^ Department of Space and Astronautical Science, The Graduate University for Advanced Studies, Hayama 240-0193, Japan.; ^32^ Department of Planetology, Kobe University, Kobe 657-8501, Japan.; ^33^ Aizu Research Cluster for Space Science, University of Aizu, Aizu-Wakamatsu 965-8580, Japan.; ^34^ Center for Data Science, Ehime University, Matsuyama 790-8577, Japan.; ^35^ Department of Physics and Astronomy, Seoul National University, Seoul 08826, Republic of Korea.; ^36^ JAXA Space Exploration Center, JAXA, Sagamihara 252-5210, Japan.; ^37^ Planetary Exploration Research Center, Chiba Institute of Technology, Narashino 275-0016, Japan.; ^38^ National Astronomical Observatory of Japan, Mitaka 181-8588, Japan.; ^39^ Geological Survey of Japan, National Institute of Advanced Industrial Science and Technology, Ibaraki 305-8567, Japan.; ^40^ Faculty of Engineering, Kindai University, Higashi-Hiroshima 739-2116, Japan.; ^41^ Faculty of Science, Niigata University, Niigata 950-2181, Japan.; ^42^ Department of Chemistry, The University of Tokyo, Tokyo 113-0033, Japan.; ^43^ Research and Development Directorate, JAXA, Sagamihara 252-5210, Japan.; ^44^ Department of Physics, Rikkyo University, Tokyo 171-8501, Japan.; ^45^ Instituto de Astrofísica de Canarias, University of La Laguna, Tenerife, Spain.; ^46^ Department of Mechanical Engineering, Kanagawa Institute of Technology, Atsugi 243-0292, Japan.; ^47^ Marine Works Japan Ltd., Yokosuka 237-0063, Japan.; ^48^ Department of Earth and Planetary Science, Tokyo Institute of Technology, Ōokayama, Tokyo 152-8550, Japan.; ^49^ School of Earth and Environmental Sciences, The University of Queensland, St Lucia, QLD 4072, Australia.; ^50^ Department of Geology, School of Earth and Environment, Rowan University, Glassboro, NJ 08028, USA.; ^51^ Lunar and Planetary Laboratory, University of Arizona, Tucson, AZ 85721, USA.; ^52^ UTokyo Organization for Planetary and Space Science, The University of Tokyo, Tokyo 113-0033, Japan.

## Abstract

The Hayabusa2 spacecraft returned to Earth from the asteroid 162173 Ryugu on 6 December 2020. One day after the recovery, the gas species retained in the sample container were extracted and measured on-site and stored in gas collection bottles. The container gas consists of helium and neon with an extraterrestrial ^3^He/^4^He and ^20^Ne/^22^Ne ratios, along with some contaminant terrestrial atmospheric gases. A mixture of solar and Earth’s atmospheric gas is the best explanation for the container gas composition. Fragmentation of Ryugu grains within the sample container is discussed on the basis of the estimated amount of indigenous He and the size distribution of the recovered Ryugu grains. This is the first successful return of gas species from a near-Earth asteroid.

## Teaser

The Hayabusa2 metal-sealed container successfully returned extraterrestrial He and Ne as a gas phase from the asteroid Ryugu.

## INTRODUCTION

Meteorites and cosmic dust provide extensive knowledge about the origin and evolution of the Solar System, but they lack geological information of the bodies where they come from and may have lost vulnerable components/phases, such as volatiles, during their atmospheric entry or after their fall to Earth. Recent advances in technology [e.g., (*1*)] enable the return of extraterrestrial materials sampled by robotic spacecraft. Some of these samples are now available and provide valuable geologic context, most notably specific information regarding the recovery site. These sample-return missions also provide the opportunity for studying previously unidentified materials that may not be represented in existing meteorite/cosmic dust collections worldwide. Last, if the reentry capsule brings the samples back from space safely, it may be possible to minimize or even eliminate detectable terrestrial contamination, weathering, destruction, and heating effects, which can occur to/in meteorites and cosmic dust during their atmospheric entry and residence on Earth’s surface (*2*–*6*). During the Hayabusa2 mission launched to the near-Earth asteroid (162173) Ryugu in 2014 by the Japan Aerospace Exploration Agency (JAXA), these effects were minimized by following protocols that include careful environment monitoring (*7*, *8*), starting with the spacecraft assembly launch and efficient, quick, and clean handling of the sample container (*9*, *10*) after the recovery. The sample container (made of aluminum alloy, with dimensions of 120 mm in diameter and 130 mm in height) was equipped within the reentry capsule and has a newly developed metal sealing system (*11*). The sample size collectable by the sampling system of Hayabusa2 is millimeters to centimeters in diameter (*10*, *12*). The metal sealing system was designed to preserve any gas released from the solid samples by adding the gas sampling interface [details of the system are described in (*7*, *11*, *13*, *14*)] to document the composition of volatiles degassed from the collected samples. Lightly retained (i.e., low-temperature released) gases, such as presolar noble gases and solar wind (SW) (*15*, *16*), released from the samples and captured by the sample container provide valuable information on the volatile compositions and on the physicochemical characteristics of the solid samples for C-type asteroids.

Hayabusa2 arrived at Ryugu on 27 June 2018 and subsequently carried out remote-sensing observations and deployments of rovers (MINERVA-II1) and a lander (MASCOT) on the asteroid (*17*). The first touchdown (TD) operation was carried out on 22 February 2019 to collect surface samples from the asteroid (*9*, *17*, *18*). After that, the small carry-on impactor (SCI) (*19*) was deployed in April 2019 to perform an asteroid-scale impact experiment and excavate subsurface material for sample collection. The second TD took place on 9 to 11 July 2019 at the site 20 m north from the SCI-made crater and permitted the collection of samples that were expected to include the impact ejecta (*9*, *17*). Each of the samples collected was stored separately in chambers A and C of the sample catcher for the first and the second TD operations, respectively (*13*). After these sampling operations, Hayabusa2 retracted the sample catcher inside the reentry capsule, which was closed and sealed on 26 August 2019 (*17*). The spacecraft left Ryugu on 13 November 2019. The reentry capsule landed on Earth on 6 December 2020 (*9*) and was recovered from the Woomera Prohibited Area (WPA), South Australia. After safety checks, the reentry capsule was transported back to a quick look facility (QLF) constructed at the WPA. The sample container was extracted from the reentry capsule at QLF. After cleaning its exterior, the sample container was connected to the vacuum line of the GAs Extraction and Analysis system (GAEA), which was developed to extract and measure volatile gases in the sample container without exposure to the terrestrial atmosphere at QLF (*13*, *14*). The vacuum line of GAEA was evacuated overnight, and the gas in the sample container was extracted and analyzed using the GAEA (*14*). The major part (~80%) of the gas was stored in four gas bottles fitted to the GAEA (Materials and Methods) before the online gas analysis for further detailed analysis.

Here, we report the composition of the gas enclosed in the Hayabusa2 sample container and subsequently analyzed on Earth and discuss possible effects of destruction and heating that the Ryugu samples might have experienced in-between sampling operation and return to Earth. The information obtained from the gases will lead to a better understanding of asteroid Ryugu, especially in combination with results obtained from ongoing and future chemical analyses of the solid samples returned from Ryugu. The results of the solid sample analyses are presented elsewhere [e.g., (*20*)].

## RESULTS

Thirty hours after atmospheric entry, the gas stored in the sample container was equilibrated with the gas analysis line of GAEA (Materials and Methods). The gas pressure of the container was immediately measured with a Pirani gauge and found to be 68 Pa. This container gas pressure was about two orders of magnitude lower than the gas pressure inside the sample container returned from S-type asteroid (25143) Itokawa by Hayabusa (~5000 Pa) (*21*). The gas inside the Hayabusa sample container was not exclusively extraterrestrial; it is likely that terrestrial atmosphere leaked into the sample container (*21*). The gas pressure inside the Hayabusa2 sample container demonstrates that the newly developed metal sealing system worked more efficiently than the Hayabusa sample container, which was sealed with double O-ring gaskets. In the Hayabusa2 metal sealing system, the softer aluminum alloy part of the container lid was pressed onto the inner edge (harder Al alloy) of the sample container with a force of ~2700 N (*7*, *11*). This metal sealing system was evaluated under several situations during its development (*11*).

**Fig. 1.**
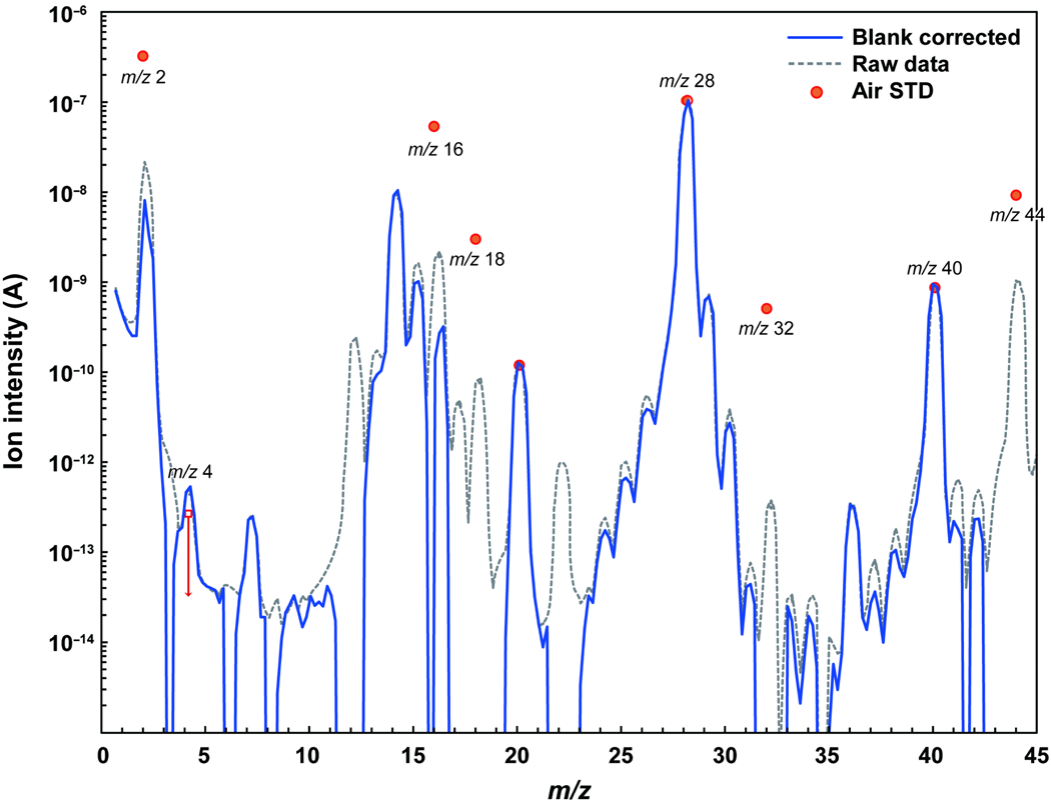
Mass spectrum of the Hayabusa2 sample container gas with the QMS installed in the GAEA. Prominent peaks from helium-4 [mass/charge ratio (*m/z*) 4], molecular nitrogen (*m/z* 28), and argon-40 (*m/z* 40) were observed in the container gas on site on 7 December 2020. The blue solid line represents the blank-corrected Ryugu gas data calculated by subtracting contributions from the instrumental blank gases, while the gray dotted line is the uncorrected (raw) measured data. The mass spectrum of the standard gas prepared from terrestrial atmosphere is also shown as red dots. The ion intensity of *m*/*z* 28 for the air was set at the same value as that of the container gas for comparison of the spectral pattern.

Most of the gas was split into several metal bottles held at (i) room temperature (bottles “NT1 to NT4”) and (ii) liquid nitrogen temperature (bottles “CR1 and CR2”) for analyses in different laboratories (Materials and Methods); the remaining fraction was analyzed on-site with a quadrupole mass spectrometer (QMS) attached to the GAEA (*14*). The QMS measurement revealed that the major species within the container gas were molecular hydrogen [H_2_; mass/charge ratio (*m*/*z*) 2], helium-4 (^4^He; *m*/*z* 4), molecular nitrogen (N_2_; *m*/*z* 28), and argon-40 (^40^Ar; *m*/*z* 40) (Fig. 1). Subsequent laboratory analyses (Materials and Methods) of the gas collected in the gas bottles confirmed that the contribution from carbon monoxide (CO) to the *m*/*z* 28 peak was negligibly small. The initial QMS spectrum is similar to the terrestrial atmosphere composition (*22*) in terms of high N_2_ and ^40^Ar abundances but different in terms of the large ^4^He and small O_2_ abundances. Clear signals from CH_4_ (*m*/*z* 16), NH_3_ (*m*/*z* 17), and CO_2_ (*m*/*z* 44) were not observed in the QMS spectra (Fig. 1); only a small amount of CH_4_ was detected during the subsequent precise and more sensitive laboratory measurements (table S1). These observations suggest that the returned Ryugu grains do not contain large concentrations of highly labile volatile components.

After the first gas collection and measurements, we observed that the inner gas pressure of the container increased by about 4.8 Pa over 2.5 hours. If the major part of the gas in the container is derived from terrestrial atmosphere, this pressure increase indicates that the degree of the leakage of the metal sealing system has not worsened after the first pressure measurement; if the leakage rate at 1.9 Pa/hour (4.8 Pa per 2.5 hours) is unchanged, the pressure measured after the first 30 hours should have been about 58 Pa. The difference between the actual pressure (68 Pa) and the estimate (58 Pa) may be due to the instantaneous intrusion of atmospheric gases into the container during the parachute deployment (*11*) and/or a slow mitigation of the leakage over time.

**Fig. 2.**
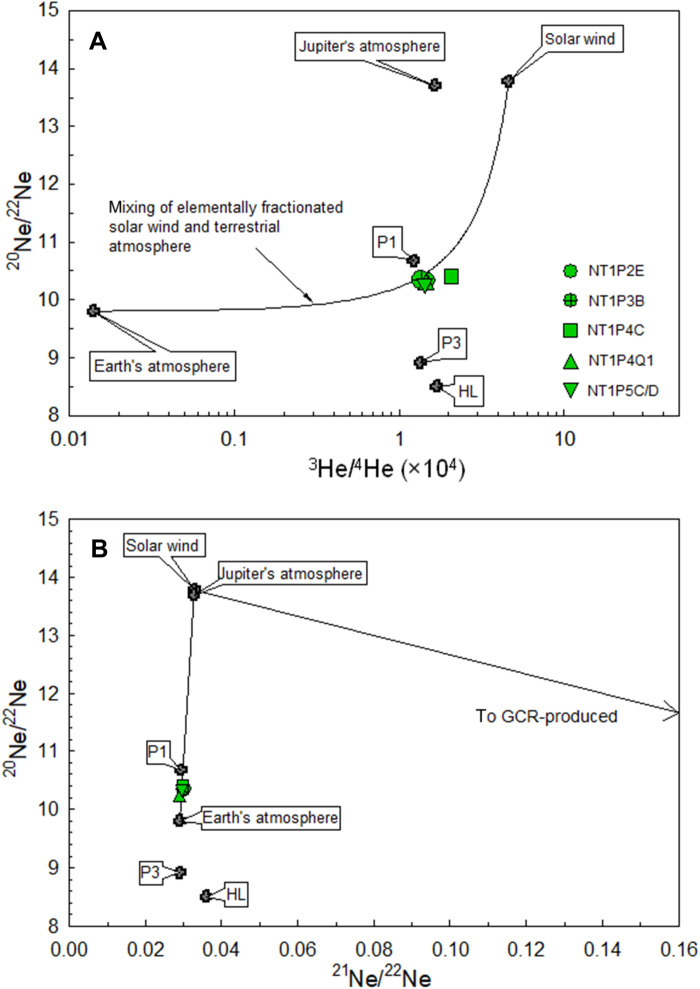
Isotopic compositions of He and Ne of the sample container. Isotopic ratios of He and Ne of the sample container were determined using gas pipettes (NT1P2E, NT1P3B, NT1P4C, NT1P4Q1, NT1P5C, and NT1P5D) separated from a gas collection bottle of NT1 (see Materials and Methods). They can be explained by mixing of solar wind (SW) and terrestrial atmosphere. The mixing line in (**A**) is calculated using the ^4^He/^20^Ne ratios of 13.1 and 8.1 for fractionated SW and terrestrial atmosphere, respectively. Neon isotopes plot on the mixing line between SW and terrestrial atmosphere (**B**). “P1 (or Q)” is a primordial gas trapped in an enigmatic (likely carbonaceous) carrier, phase Q (*15*). “P3” and “HL” are presolar gas components residing in presolar nanodiamond grains (*15*). A mixing line between SW and galactic cosmic ray (GCR)–produced Ne is also shown. Data sources are as follows: (*23*–*25*) for terrestrial atmosphere; (*26*) for Jupiter’s atmosphere; (*15*) for P1, P3, and HL gases; and (*16*) for SW.

**Fig. 3.**
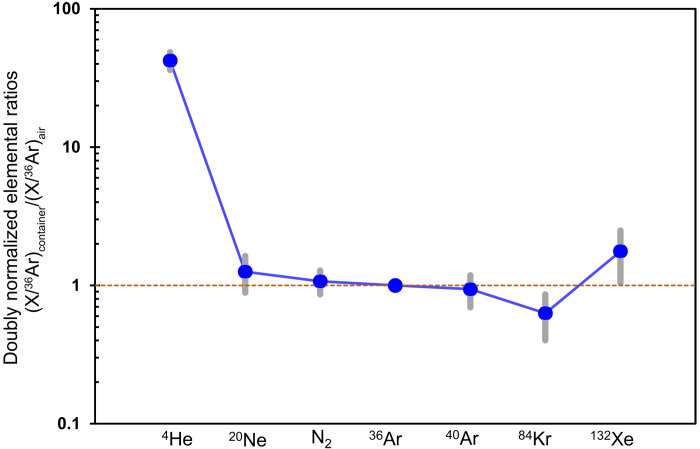
Elemental compositions of noble gases and nitrogen in the Hayabusa2 sample container normalized to Earth’s atmospheric composition and ^36^Ar. Mass spectrometry analyses for the gas collection bottle were performed at several laboratories. Uncertainties (1σ) are shown as the light gray bars. A clear excess in ^4^He was observed compared with Earth’s atmospheric composition (*22*). Doubly normalized elemental ratios, (X/^36^Ar)_container_/(X/^36^Ar)_air_, for N_2_ or isotope X (X = ^4^He, ^20^Ne, ^36^Ar, ^84^Kr, or ^132^Xe).

To gain further knowledge on the gas compositions, the volatile contents in one of the gas collection bottles (NT1) were analyzed in nine gas pipettes at seven laboratories that are part of the Hayabusa2 Initial Analysis volatile subteam (Materials and Methods). The measured gas compositions are in good agreement among the laboratories within 1 and 3% for isotopic and elemental ratios, respectively (table S1), proving the validity of our analyses. The weighted arithmetic mean value of measured ^3^He/^4^He ratios of the container gas is 1.428 ± 0.010 × 10^−4^ (Fig. 2A and table S1), ~100 times higher than that of Earth’s atmosphere (1.34 × 10^−6^) (*23*). Moreover, the Ne isotopic composition (^20^Ne/^22^Ne = 10.3427 ± 0.0055 and ^21^Ne/^22^Ne = 0.02980 ± 0.00015) (Fig. 2A and table S1) also differs from the atmospheric composition (^20^Ne/^22^Ne = 9.8 and ^21^Ne/^22^Ne = 0.0285) (*24*, *25*), beyond the uncertainties obtained by the weighted average of measurements from the different laboratories. The isotopic ratios and relative abundances of Ar, Kr, Xe, and N are essentially the same as those of Earth’s atmosphere (Fig. 3), although ^40^Ar/^36^Ar ratios (268.4 to 284.7) and the δ^15^N [−16 to −13 per mil (‰)] are slightly different from those of the terrestrial atmosphere (table S1), suggesting that there is a small contribution of terrestrial components with fractionation favoring in lighter isotopes. These results indicate that the container gas is a mixture of the He and Ne released from the Ryugu grains and the terrestrial atmosphere, which was most likely introduced by a small leak (1.9 to 2.3 Pa/hour) in the metal sealing system (*11*).

## DISCUSSION

### Timing and contribution of the terrestrial atmosphere leakage

The QMS data obtained online in the GAEA (Fig. 1) imply that the container gas consists mainly of terrestrial atmosphere with a substantial amount of extraterrestrial He, i.e., gas released from the Ryugu grains. The 68 Pa of the sample container pressure measured 30 hours after atmospheric entry corresponds to a leak rate of ~2 Pa/hour for the atmospheric N_2_. If this was the case, a peak of O_2_ (*m*/*z* 32) should also be observed considering the high *m*/*z* 28 (N_2_) peak but not observed (Fig. 1). It is likely that O_2_ originating from the terrestrial atmosphere was adsorbed onto the inner surface of the sample container (Al alloy) (*7*, *11*) and the vacuum line of GAEA (stainless steel) (*14*) due to its higher affinity to metal and/or decomposed by ionization with the ion pressure gauge [as was observed when air-standard gas was measured during rehearsal operations of GAEA: (*14*)]. Ryugu grains are highly porous (*10*) and have large surface areas, and hence it is likely that adsorption onto and/or reaction with the Ryugu grains is also a possibility; assuming that the 68 Pa in a 200-cm^3^ volume of the container was originally composed of terrestrial N_2_ and O_2_ molecules, the maximum amount of oxygen adsorbed on Ryugu grains is estimated to be ~1 × 10^−6^ mol (~40 μg), which is 1 × 10^−5^ times smaller than the total sample mass (~5.4 g). Terrestrial H_2_O molecules were also expected to contaminate the detected gas, but their amount is negligible and could be adsorbed on the grains as with O_2_; an expected amount of H_2_O introduced by a small leak in the metal sealing system is 4 × 10^−5^ mol (calculated by assuming a temperature of 20°C and 50% humidity), which is ~1 × 10^−4^ of the total sample mass.

Between the sample container closing operation and its arrival at Earth, Ryugu grains should have experienced fragmentation and fracturing due to vibration and acceleration/deceleration during the subsequent operations, such as target markers/MINERVA-II2 deployments and the orbital controls for 1 year and 3 months (*17*). During this period, Ryugu grains would have continued to release gases. However, the presence of extraterrestrial gases in the sample container suggests that the small leakage did not occur in outer space but started after atmospheric entry, possibly caused by an instant opening of the metal sealing due to shock during parachute deployment (*11*). This deployment shock could have changed the original seal surface after instant opening. The later seal surface might intersect the original seal surface and enclose Ryugu grains between the seal surface, which could have caused the continuing leakage observed at the QLF. The possibility of grain incorporation onto the seal surface should be investigated as the future work.

If the container had leaked in space, most of the gas from the Ryugu grains, particularly small atoms and molecules, such as He, would have been lost to space, and the gas that remained in the sample container would have mainly been released after atmospheric entry. The nominal mission specification of the container allowed for a leak of a total of 1 Pa over 100 hours, an estimated duration from the capsule landing to the gas recovery, at the atmospheric pressure (*11*). This specification was set for 0.1 g of the returned sample, considering volatile release from the samples that would have included SW components. Considering the total sample mass of 5.4 g, the requirement can be relaxed to ~50 Pa of air for a 5.4-g sample mass. Therefore, the air leakage of 68 Pa before gas sampling is slightly larger than the specification but small enough to permit the discussion below.

### Origin of the He and Ne stored in the sample container

To evaluate the origin of He and Ne in the container, we modeled the container gas composition using a two-component mixing of Earth’s atmosphere and an extraterrestrial component (Materials and Methods). We assumed elemental fractionation of two components between He and Ne without any isotopic fractionation. This elemental fractionation could have occurred in the terrestrial atmosphere during the leakage and in the extraterrestrial gases during incorporation into and/or outgassing from the Ryugu grains. The least-square fitting calculation (Materials and Methods) revealed that mixing of fractionated SW and terrestrial gases best explains the observed noble gas composition. In comparison to the terrestrial atmosphere, other components such as a primordial noble gas component P1 (or Q) present mainly in enigmatic (most likely carbonaceous) material, presolar noble gas components (HL and P3) carried by presolar diamonds, and the Jupiter atmosphere (i.e., gases assumed to be the protosolar nebula composition) (*15*, *26*) were also tested (fig. S1). We found that the mixing using these components cannot reproduce the observed compositions.

The ^4^He/^20^Ne ratios obtained (for the container gas composition calculated as the weighted arithmetic mean value) from our model (Materials and Methods) are 13.1 ± 0.6 and 8.1 ± 0.3 for fractionated SW and terrestrial atmosphere, respectively (fig. S1). The ^4^He/^20^Ne of 13.1 is lower than the unfractionated SW (656) (*16*) as observed in many solar gas–rich meteorites (*27*) and Itokawa particles (*28*). This elemental fractionation can be explained by preferential loss of the highly labile ^4^He form and/or elemental fractionation during SW implantation onto Ryugu grains’ surface. The ^4^He/^20^Ne for fractionated terrestrial atmosphere (8.1) is consistent with elemental fractionation, favoring smaller elements due to preferential intrusion of atmospheric He through pores in the metal sealing surface. Contributions from the terrestrial atmosphere are calculated to be ~70 and ~80% for ^4^He and ^20^Ne, respectively. By subtracting these contributions, we determine the SW-derived ^4^He and ^20^Ne abundances released from the Ryugu grains to be 8 × 10^−6^ and 6 × 10^−7^ cm^3^ STP (standard temperature and pressure; 0°C and 1 bar), respectively.

### Mechanism liberating the SW gases from Ryugu grains

It is important to discuss how the gas in the container was released from Ryugu grains, since knowing the mechanism would allow better understanding the initial (right after the collection) condition of the grains collected from the Ryugu surface (e.g., the original size distribution and shapes) and their volatile budgets. Potential mechanisms for the release of SW gases from the Ryugu grains in the sample container after the sampling from the asteroid surface include thermal and/or mechanical effects, such as particle fragmentation and heating during atmospheric entry.

First, we evaluate isotropic (i.e., uniform destruction, not the surface pulverization) mechanical fragmentation of cubic grains (details are presented in Materials and Methods). The actual shapes of Ryugu samples are diverse: They can be blocky, irregular, and spherical (*9*, *10*). The assumption of a cubic shape is reasonable and allows discussing, in a simple way, the issue in a first-order estimation, as shown by comparison between cubic and spherical models (Materials and Methods).

**Fig. 4.**
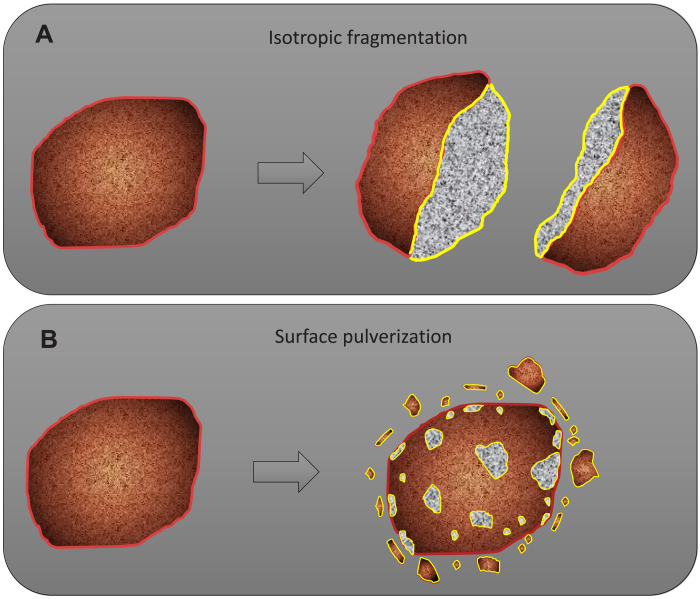
Schematic diagram of the fragmentation and pulverization of the Ryugu grains. The original grain surfaces are assumed to have been exposed to SW that is concentrated within the ~50-nm-thick uppermost layer (red line). Isotropic fragmentation (**A**) and surface pulverization (**B**) generate a fresh cross-section surface of the SW layer (yellow line). The surface pulverization seems consistent with the presence of many powdery samples sticking on the inner surface of the sample container (*10*, *32*).

The maximum release of the SW-derived He during isotropic fragmentation was estimated by assuming that (i) the Ryugu sample was originally three 1-cm-sized cubes [this was the maximum obtainable size in the Hayabusa2 sampler (*10*, *12*)] with a density of 1.8 g cm^−3^ (*29*), (ii) the SW had been implanted into the outermost 50-nm-thick layer of the original 1-cm samples (*30*, *31*), and (iii) the SW gases were released from the newly exposed surfaces by isotropic fragmentation of the 1-cm-sized cubes until the cube size decreases to 0.13 cm (Fig. 4A and fig. S2). The terminal size of 0.13 cm is determined on the basis of the actual median size of the grains recovered (*10*). In this isotropic fragmentation case, the total fresh surface area is calculated to be ~3 × 10^−3^ cm^2^ (Materials and Methods). If SW-^4^He of 8 × 10^−6^ cm^3^ STP was released from the surface area of ~3 × 10^−3^ cm^2^, the surface concentration of SW-^4^He in Ryugu grains was ~3 × 10^−3^ cm^3^ STP cm^−2^, corresponding to ~1 × 10^−2^ cm^3^ STP-^4^He g^−1^ in a 1-cm-sized cube. This is much higher than the actual ^4^He concentrations of ~1 × 10^−4^ to 4 × 10^−3^ cm^3^ STP g^−1^ of the Ryugu solid samples (*19*). To be consistent with the SW-^4^He concentrations in Ryugu grains, more efficient destruction cases, such as pulverization of SW-implanted grain surface, are required (Fig. 4B).

In the case of surface pulverization, only smaller particles will be produced, which may explain the size distribution of the Ryugu grains enriched in smaller fractions compared to that of the Ryugu surface boulders (rocks greater than 0.3 m in diameter) (*10*). Assuming that all of the original (i.e., before surface pulverization) Ryugu grains are 0.13-cm-sized cubes with a ^4^He concentration of 4 × 10^−3^ cm^3^ STP g^−1^ (the highest value observed in a sample recovered from the first TD site) (*20*), the pulverization of merely several percent of the total surface areas of the Ryugu grains can explain the measured amount of SW-derived ^4^He in the sample container (Materials and Methods). In this case, the present size distribution of the Ryugu samples has been largely unchanged, since the collection on the asteroid and the powdery materials observed inside the chambers of the Hayabusa2 sample catcher could be the fragments resulting from surface pulverization (*10*, *32*). Even if a sphere is assumed as the initial particle shape instead of the cubic shape, the result does not change in this first-order estimation.

We deem temperature increase during atmospheric reentry as unlikely to be the cause of the gas liberation. Previous studies found that SW-rich meteorites and lunar samples released ~0.5% of the total ^4^He at 80°C, with the exception of one sample (*33*, *34*). Other extraterrestrial components can be released at higher temperatures (*15*). In contrast, the maximum temperature at Ryugu’s TD site is about 70°C (*35*), yet the temperature monitor installed on the sample container in the Hayabusa2 reentry capsule has never experienced temperatures above 65°C (*10*). In addition, thermal release would work more efficiently for the lighter He than for Ne, but the calculated ^4^He/^20^Ne of 13.1 for noble gases released from the Ryugu grains is lower than those of SW (656) (*16*) and an SW-rich Ryugu sample (~50) (*20*).

Continuing efforts since before the launch of the Hayabusa2 spacecraft have allowed us to bring back the asteroidal gas samples from Ryugu to Earth and have given us an opportunity for measurements. The measured composition of the gas stored in the Hayabusa2 sample container indicates that the Hayabusa2 mission has successfully returned solid materials from the surface and subsurface of Ryugu to Earth, with little mechanical and thermal degradation. In this regard, the Hayabusa2 sample container is a treasure box from a near-Earth asteroid.

## MATERIALS AND METHODS

### Gas sampling and on-site measurement at the QLF in WPA

The reentry capsule of Hayabusa2 recovered from the WPA was transported to QLF to extract the sample container for the on-site sampling and measurement of gas species stored in it. After the safety check and cleaning operations, the sample container was connected to the vacuum line of GAEA through the gas sampling interface of the container (*14*). The vacuum line other than the sample container was evacuated and heated for 8 hours before the subsequent operations. The bottom of the sample container, made of aluminum alloy, was pierced using a tungsten carbide needle (*14*) in the vacuum line of the GAEA to extract the gas. The gas extracted from the container was equilibrated in the vacuum line and measured its pressure with a Pirani gauge. The inner gas pressure of the sample container was calculated to be 68 Pa based on the volume ratio of the sample container (the inner volume: ~200 cm^3^) and the vacuum line (79 cm^3^ including the Pirani gauge) (*14*). About 80% of the gas in the sample container was collected to four gas bottles (NT1 to NT4, each bottle has a volume of 750 cm^3^) (*14*) at room temperature 30 hours after atmospheric entry. The gas split in a volume (16 cm^3^) (*14*) before the collection into the gas bottles, corresponding to ~4% of the container gas, was kept for the QMS measurement to determine the gas composition. The remaining gas was trapped into two gas bottles, CR1 and CR2, at liquid nitrogen temperature (*14*). Blank gases of the vacuum line were collected to NT5 and CR3 at room temperature and liquid nitrogen temperature, respectively. Further details are presented in (*14*).

### Preparation of the gas samples from the gas collection bottles

The gas collection bottles (NT1 to NT5 and CR1 to CR3) were brought back to the curation facility of JAXA on 8 December 2020 along with the sample catcher/container (*10*). NT5 and CR3 are bottles for the instrumental blank runs of GAEA. Details and results of measurements for CR1 to CR3 will be reported elsewhere. The sample gas pipettes for the volatile compound analysis were prepared first, on 9 December 2020, from the gas collection bottles of NT1 and NT5 containing the container gas and the blank gas, respectively (pipettes NT1P1A, NT1P1B, NT5P1C, and NT5P1D), because volatile compounds may change their chemical structure in the gas collection bottles, which may occur through interactions with hydrogen and other gas species from the gas bottles. The gas pipettes for noble gas and nitrogen analyses were prepared later, on 20 January 2021 (pipettes NT1P2E, NT1P2F, NT1P3A, NT1P3B, NT1P4C, NT1P4Q1, NT5P2G, NT5P2H, NT5P3D, and NT5P3Q2). The rule for naming the gas pipettes is as follows, taking “NT1P2E” as an example: The first three characters of “NT1P2E” denote the name of the gas collection bottle, here NT1. The following “P2” indicates that the gas pipette was aliquoted in the second pipetting operation. The “E” is the name of the metal vessel.

The sample gas pipettes were distributed to seven laboratories, Kyushu University, Ibaraki University, Université de Lorraine CRPG-Nancy, Eidgenössische Technische Hochschule (ETH) Zürich, Washington University, Tokyo Institute of Technology (TITECH), and Japan Agency for Marine-Earth Science and Technology (JAMSTEC). These laboratories belong to the volatile subteam of the Hayabusa2 Initial Analysis. The gas sample pipettes (NT1P1A, NT1P1B, NT1P2E, NT1P2F, NT1P3A, NT1P3B, NT1P4C, NT5P1C, NT5P1D, NT5P2G, NT5P2H, and NT5P3D) were measured within 1 month at the laboratories.

Exceptionally large amounts (~20 times larger than the amount expected) of atmospheric gases were found in sample gas pipette NT1P3A due to leakage through the valves placed on the pipette. Hence, two other pipettes, NT1P5C and NT1P5Q1, were prepared later, on 14 May 2021. These two pipettes were measured along with the previously prepared pipettes NT1P4Q1 and NT5P3Q2. There are higher abundances of noble gases in NT1P4Q1 compared to NT1P5C and NT1P5D due to outgassing from the pipette and/or its vacuum line during the 4-month storage period before analysis. Comparison between the NT1P5C/NT1P5D and other pipettes prepared earlier (e.g., NT1P2E) shows that they are in good agreement with each other. This indicates that the gas collection bottles (NT1 to NT5) preserved the native compositions of the gas stored in the sample container.

### Analysis procedures of the volatile compositions in the sample gas pipettes

Volatile compositions in the sample gas pipettes were measured through the following procedures at each laboratory.

At Kyushu University: The gas pipette (NT1P4C) and the blank pipette (NT5P3D) were connected to the purification line of the noble gas mass spectrometry system connected to a modified mass spectrometer (MM5400, Micromass). The gas in each pipette was expanded to the purification line and was purified with titanium (Ti)–zircon (Zr) getters and an aluminum-zirconium getter (NP-10, SAES). Heavy noble gases (argon, krypton, and xenon) were separated using charcoal traps to introduce them separately from helium and neon into the mass spectrometer. Blank levels determined by NT5P3D are shown in table S1. Details of analytical procedures and descriptions of the instruments are described in (*36*).

At CRPG-Nancy: The blank (NT5P2G) and sample (NT1P2E) pipettes were first connected to the purification line of the Thermo Scientific Helix MC Plus for the analysis of He, Ne, Ar, Kr, and Xe gas abundance and isotope ratio determination. A fraction of the gas from the bottles was admitted to the purification line and purified by being passed through an in-line Ti sponge getter held at 650°C. The gas was then further purified by exposure to a further two hot (550°C) and two cold (50°C) Ti sponge getters before the heavy noble gases were separated from He and Ne by being condensed on to a charcoal finger at −196°C. Helium and Ne were separated by condensing Ne onto a liquid He cooled cryotrap at −239°C. Last, Kr and Xe were separated from Ar by being trapped on a quartz glass cold finger at −196°C. The isotopes of He, Ne, Ar, Kr, and Xe were then analyzed on the Helix MC Plus following the same procedure as in (*37*).

Following the initial analysis of the noble gas isotopes using the Helix MC Plus, the blank and sample bottles were then connected to the purification line of the Noblesse HR (Nu Instruments) noble gas mass spectrometer for Ne, Ar, and N_2_ abundance and isotopic analyses. Two analyses of the residual gas in the “blank” and “sample” bottles were performed. Noble gases were purified using two hot (600°C) Ti sponge getters and two cold (room temperature) SAES Ti-Al getters. Argon was separated from neon by adsorption onto a charcoal finger at −196°C. Nitrogen was purified in a Pyrex and quartz glass line using a copper oxide (CuO) furnace cycled between 450° and 900°C and a U-shaped cold trap held at −180°C. The three isotopes of Ne and Ar and the three isotopologues of N_2_ were analyzed sequentially using multicollection (*38*, *39*).

At Ibaraki University: The gas pipette (NT1P2F) and the blank pipette (NT5P2H) were connected to the mass spectrometry line (*40*) for nitrogen isotope analyses. Sample gas in the bottle was first expanded to the adjacent volumes, which collectively correspond to approximately 83% of the volume of the bottle, and then it was further separated by valves. Fractions of the sample gas were successively analyzed. The gas was first introduced to the vacuum line designated to reduce combustible compounds, converting them to carbon dioxide (CO_2_), water (H_2_O), and N_2_. Sample gases mixed with approximately 1 torr of pure oxygen, in contact with a platinum foil, were heated at 800°C. After absorbing the remaining oxygen gas by CuO, CO_2_ and H_2_O were removed by a cryogenic trap cooled at liquid nitrogen temperature. After pressure adjustment, the sample gas was lastly introduced to a QMS (QMA420, Balzers) operating in static mode for nitrogen mass spectrometry [see (*40*, *41*) and references therein for further procedural details and analytical performances]. Standard gas measurements were performed six times during the Hayabusa2 sample gas analytical session. The reproducibility for the nitrogen isotope ratio was 0.4‰ (1σ) for 60-pmol standard N_2_ gases. The system blank for N_2_, including all purification procedures, measured during this session was 1.3 pmol.

At TITECH: The gas pipette (NT1P1A) and the blank pipette (NT5P1C) were connected to a vacuum line for extracting volatile components coupled with a gas chromatography–QMS (GC-QMS) system (7820A/5977D GC-MSD, Agilent Technologies Inc., USA) at TITECH. The gas in each pipette was introduced into the vacuum line by diffusion, and volatile components were cryogenically collected in a trap at −196°C. Then, the collected volatile components were introduced by a helium stream into the GC-QMS system to analyze the composition of the volatile components methane (CH_4_), ethane (C_2_H_6_), CO, CO_2_, H_2_O, N_2_, and O_2_.

At JAMSTEC: Methane and ethane in the gas pipette (NT1P1B) and blank pipette (NT5P1D) were purified by purge-and-trap gas chromatography before the introduction into an isotope ratio mass spectrometer (MAT253, Thermo Fisher Scientific, Bremen, Germany) (*42*, *43*). Neither methane nor ethane was detected (cf. detection limit = 6 pmol), implying that abundances of methane and ethane in the Ryugu grains were not higher than 90 nmol g^−1^, calculated barometrically from sample size and volume of the vacuum line. H_2_ was also analyzed in a similar manner described in (*44*), but not detected.

At Washington University: A gas pipette (NT1P3B) isolated by two sequential all-metal Swagelok valves was connected to the sample system equipped with MKS Baratron (133 Pa range). The gas aliquot between Swagelok valves was further expanded to a purification line. The latter consisted of SEAS getters: two NP-10, one D50, and a small Ti sublimation pump. Noble gases were separated cryogenically and sequentially admitted to an in-house built 21-cm-radius 90° magnetic sector mass spectrometer equipped with a high-transmission (>90%) ion source operated at 3 kV acceleration voltage for Kr, Xe, and Ar and at 4 kV for light noble gases. This configuration results in low memory (build-up of previously analyzed gas) and long (>1 hour for Xe and Kr) useful ion counting time. Instrumental mass discrimination is extremely small (~0.06%/Da for Xe) and stable due to the absence of a magnetic field in the ion source region.

At ETH Zürich: Two sets of pipettes were allocated by JAXA, first one sample pipette (NT1P3A) and then a set of sample and blank pipettes (NT1P4Q1, NT1P5C, NT1P5D, and NT5P4D). Each set was connected to the gas cleaning and separation line attached to the custom-built mass spectrometer “Albatros” (*45*) via another pipette (~1 cm^3^). Gases were cleaned for reactive gas molecules with several commercial (SAES) getters held at different temperatures between 20° and 350°C [see (*45*) for details]. The cleaned gases were separated by charcoals held at −196° and −125°C, respectively, and measured in three fractions (He-Ne, Ar, and Kr-Xe, with Ar and Kr-Xe in the other respective phases being corrected for). We used a custom-built sector field mass spectrometer Albatros equipped with a Baur-Signer source, providing linearity over a large dynamic range, a multiplier run in ion counting mode, and a Faraday cup. Electrons accelerated by 45 V ionized the gases [cf. (*45*) for further references]. Container and blank gas concentrations and isotopic compositions were determined by comparing them with precisely known amounts of calibration gas mixtures (*45*).

### Least-square fitting calculation of the elementally fractionated gases in the Hayabusa2 sample container

We assume that the noble gases in the sample container are a mixture of extraterrestrial and terrestrial atmospheric gases that are fractionated only in their elemental ratios but not in their isotopic ratios. Isotopic ratios of ^3^He/^4^He are fixed for the measured, extraterrestrial, and terrestrial gases and were used as the most reliable parameters for the calculation: The obvious difference between the measured and terrestrial values was observed, and isotopes with smaller differences in mass and atomic size should be less fractionated than elements with larger mass and size differences.

The measured ^4^He/^20^Ne and ^3^He/^4^He ratios of the container gas should be explained by mixing of air and an extraterrestrial component with fixed ^3^He/^4^He ratios and variable ^4^He/^20^Ne due to elemental fractionation (e.g., for SW gas as shown in fig. S1A). We first tested this model with the SW as the extraterrestrial component, because it is expected that SW is the most abundant and most common in asteroidal surface materials and most enriched in He compared to other known extraterrestrial components (*14*, *15*). Among a number of ^4^He/^20^Ne ratios for the fractionated air and SW, we determined the most probable ^4^He/^20^Ne ratios as follows. The ^36^Ar/^20^Ne ratios of air and SW are expected not to be significantly fractionated, because their mass (and atomic radius) difference is smaller than that between He and Ne. The ^36^Ar/^4^He and ^3^He/^4^He ratios of the container gas should be explained by a mixing of fractionated air and SW (fig. S1B). Therefore, using the fixed ^36^Ar/^20^Ne ratios of air and SW, we determined the ^4^He/^20^Ne ratios of the fractionated air and SW by the least-square method to minimize the difference in the ^36^Ar/^4^He-^3^He/^4^He ratios between the container gas and the calculation (i.e., to best explain the ^36^Ar/^4^He-^3^He/^4^He mixing relation). We used the ^36^Ar/^20^Ne ratios of 1.91 (*24*) and 0.024 (*16*) for air and SW, respectively, to obtain ^36^Ar/^4^He ratios (fig. S1B). The composition of the container gas (table S1) used for the mixing calculation was calculated on the basis of the weighted arithmetic mean method by using the dispersions (1/σ^2^) of ^3^He/^4^He ratios as the weighing factors. The obtained ^4^He/^20^Ne ratios of the fractionated SW and air were 13.1 ± 0.6 and 8.1 ± 0.3, respectively. These values can explain the mixing observed in the ^4^He/^20^Ne-^36^Ar/^20^Ne and ^4^He/^20^Ne-^22^Ne/^20^Ne plots (fig. S1, C and D). The elemental fractionation expected for SW and air would involve isotopic fractionation between ^3^He and ^4^He too, but it does not greatly affect the result of this calculation. Rather, the elemental ratio of ^36^Ar/^20^Ne for the fractionated air, which was assumed to be constant, could be more variable and cause larger uncertainties than isotopic ratios: A 5% lower ^36^Ar/^20^Ne ratio for the fractionated air (the maximum value expected from the measured N_2_/^36^Ar ratio) would result in ~25% higher and ~5% lower ^4^He/^20^Ne ratios of the fractionated SW and air, respectively. This degree of uncertainty in the estimation of the fractionated SW and air compositions does not affect the following discussion about the gas release mechanism.

We also tested other end members as a counterpart of Earth’s atmosphere; Jupiter’s atmosphere is considered to represent the protosolar disk gas (*26*), and planetary and presolar gases (P1, P3, and HL gases) (*15*). However, we found that they cannot reproduce the mixing relation as well as SW (e.g., fig. S1, E and F).

### Estimate of the fresh SW layers generated by isotropic fragmentation of large particles

To estimate the degree of destruction of the Ryugu samples after the collection operations on the asteroid, it might be informative to compare the size distributions of the Ryugu samples in the container (*10*) with those of Ryugu surface boulders and gravels (*46*). However, it is impossible, because only the total recovered mass and the slopes (i.e., power index of the grain size distribution) are available restraining conditions for the initial (i.e., before fragmentation) size distribution of the Ryugu samples: To calculate the degree of destruction quantitatively, we have to define the minimum and the maximum sizes (the latter significantly affects the result) of the initial Ryugu samples, but the information is impossible to obtain. Therefore, we cannot make such quantitative calculation, and we tried to estimate the maximum release of ^4^He by grain destruction as follows.

Ryugu samples stored in the sample catcher are about 5.4 g in total weight with a bulk density of 1.8 g cm^−3^ (*29*) and have a size distribution with the particle size mode of around 1.3 mm [calculated for the size range between ~0.5 and ~8 mm based on (*10*, *47*)]. We assumed that these grains formed through isotropic fragmentation of larger cube-shaped grains with a side length of *D*_p_ (corresponding to the particle diameter). *D*_p_ was assumed to be 1 cm, which is the maximum collectable dimension for grains that enter the sample catcher through the sampler horn (*10*, *12*), corresponding to three cubes of Ryugu samples with the total mass of 5.4 g as the initial condition. This assumption would provide the maximum fresh surface area (i.e., the most disruptive case for isotropic fragmentation). We also assumed that SW had been implanted in all six faces of the cubes with an implanted layer thickness of 50 nm (*30*, *31*).

In this calculation, we express the fragmentation steps using a parameter “*n*.” At a fragmentation step *n*, a cube is broken into eight cubes with a side length of *D*_p_/2*^n^*; for *n* = 1, the original cube (length *D*_p_ on side) is broken into eight cubes, each having three SW-exposed surfaces that yield six fresh surfaces of the SW-exposed layer. The edge of the newly generated cubes (cross sections of the SW-exposed layers) releases SW gases (fig. S2). For *n* = 2, each of the eight cubes generates one cube with three SW-exposed surfaces yielding six fresh surfaces, three cubes with two SW surfaces yielding four fresh surfaces, three cubes with one SW surface yielding two fresh surfaces, and one cube with zero SW surface yielding zero fresh SW surface. Table S2 summarizes the result of the calculation.

If the sample is assumed to be spherical (1 cm^3^ in volume with 1.24 cm diameter) rather than 1-cm-sized cubic, the cumulative surface area for *n* = 2 is 3.19 × 10^−4^ cm^2^, almost the same as that for the cubic shape (3.60 × 10^−4^ cm^2^). However, there is variety in the shapes of the fragments for *n* > 1 (fig. S3), which makes the calculation intricate and complicated.

The original 1-cm-sized cubes are fragmented into 0.13-cm-sized cubes at *n* = 3 (table S2). Starting from three original cubes, the number of the cubes formed through the isotropic fragmentation with *n* = 3 is 1550, and the cumulative area of the fresh SW layer exposed by fragmentation is calculated to be 3 × 10^−3^ cm^2^.

### Expected fraction of pulverized surface area

Before the gas collection in QLF, numerous kinetic impacts and vibrations during the Earth return operation should have occurred, which could have caused surface deformation (i.e., pulverization) of Ryugu grains rather than isotropic fragmentation. We estimated the fraction of the pulverized surface area to account for the observed SW-derived ^4^He abundance in the sample container. It was assumed that 1000 cube-shaped Ryugu grains with 0.13 cm in length (the median size of the recovered grains in the range of 0.5 to 8 mm) (*10*), corresponding to ~5.4 g of total mass for the density of 1.8 g cm^−3^ (*29*), were originally (before pulverization) present in the sample container. It was also assumed that these original grains contain SW-^4^He homogeneously in the outermost 50-nm-thick layers. Assuming a bulk concentration of 4 × 10^−3^ cm^3^ STP g^−1^ for a Ryugu solid particle (*20*), the SW-^4^He density should be 8 × 10^20^ atoms cm^−3^, from which the surface SW-^4^He density of 9 × 10^13^ atoms cm^−2^ is obtained. This SW-^4^He surface density leads us to conclude that grain surface pulverization to form a fresh surface area of 2 × 10^−3^ cm^2^ is required for each Ryugu cube to explain the total amount of SW-^4^He released inside the sample container. The surface area of 2 × 10^−3^ cm^2^ corresponds to pulverization of 2% of the surface area from each 0.13-cm-sized cube or to the production of 2000 fragments with 10 by 10 μm^2^ area and <50 nm thickness. The latter case (generation of fine dust from the grain surface) seems to be more realistic. Such powdery samples were observed in the sample catcher and may have contributed to the release of SW gas from the returned particles.

## Supplementary Material

20221020-1
