# Supplementary material for: First asteroid gas sample delivered by the Hayabusa2 mission: A treasure box from Ryugu
Source: Sci Adv. 2022 Nov 16;8(46):eabo7239. doi: 10.1126/sciadv.abo7239 (PMC11627213; doi:10.1126/sciadv.abo7239)
Supplement: Supplementary file 2 — Figs. S1 to S3 Tables S1 and S2 [file sciadv.abo7239_sm.pdf]

Science Advances
AAAS

Supplementary Materials for

First asteroid gas sample delivered by the Hayabusa2 mission: A treasure box from Ryugu

Ryuji Okazaki *et al.*

Corresponding author: Ryuji Okazaki, okazaki.ryuji.703@m.kyushu-u.ac.jp

Sci. Adv. **8**, eabo7239 (2022)
DOI: 10.1126/sciadv.abo7239

This PDF file includes:

Figs. S1 to S3
Tables S1 and S2

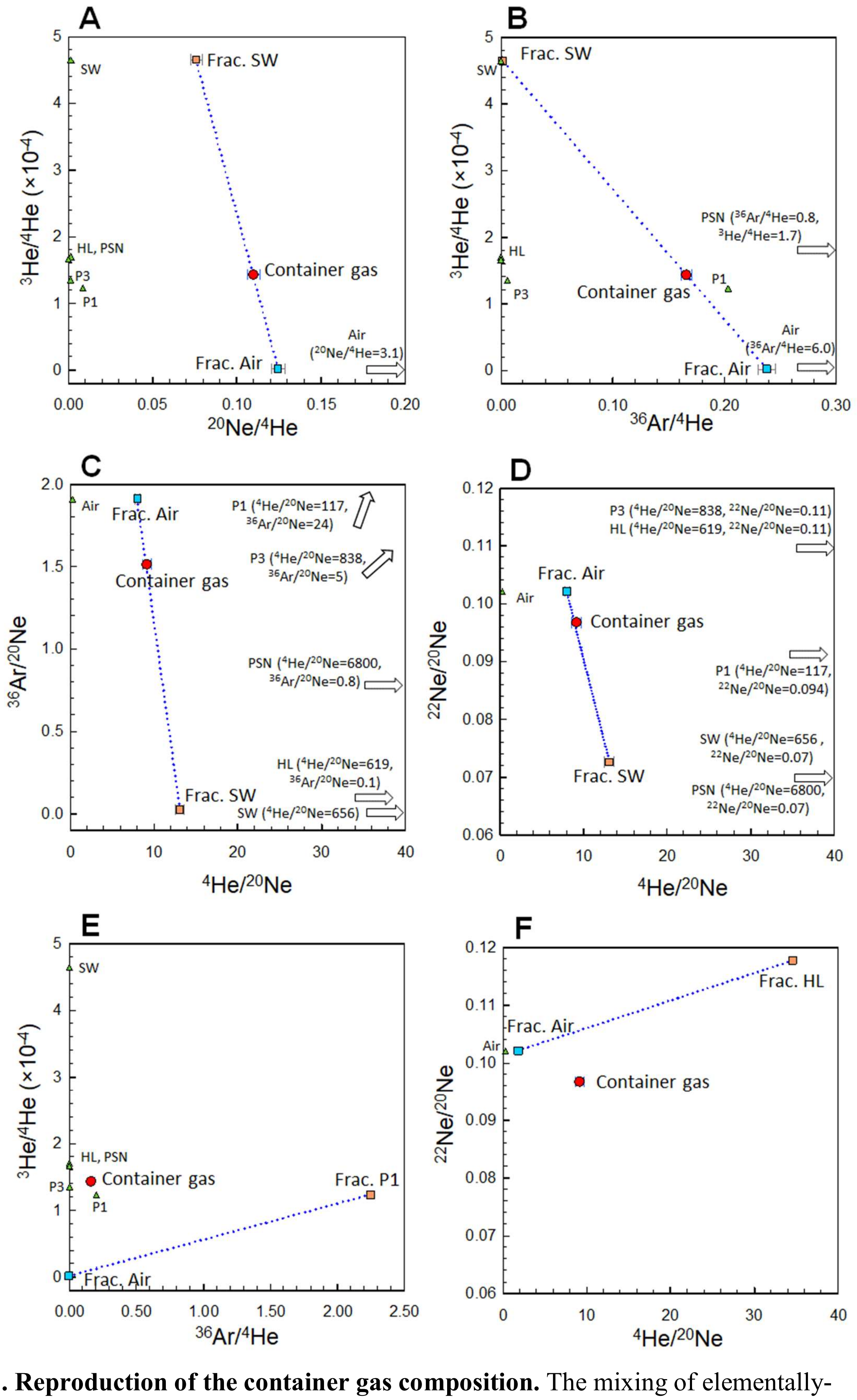

Fig. S1. Reproduction of the container gas composition. The mixing of elementally-

fractionated solar wind (Frac. SW) and terrestrial atmosphere (Frac. Air) explains the observed isotopic and elemental ratios of He and Ne (Figs. S1A–D). Other noble gas components are also plotted for comparison. Two invalid cases are shown in Figs. S1E and S1F for the fractionated P1 and HL components, respectively. PSN denotes Proto Solar Nebula inferred from the Jupiter's atmosphere (*26*). Data sources: *15, 16, 23–26*.

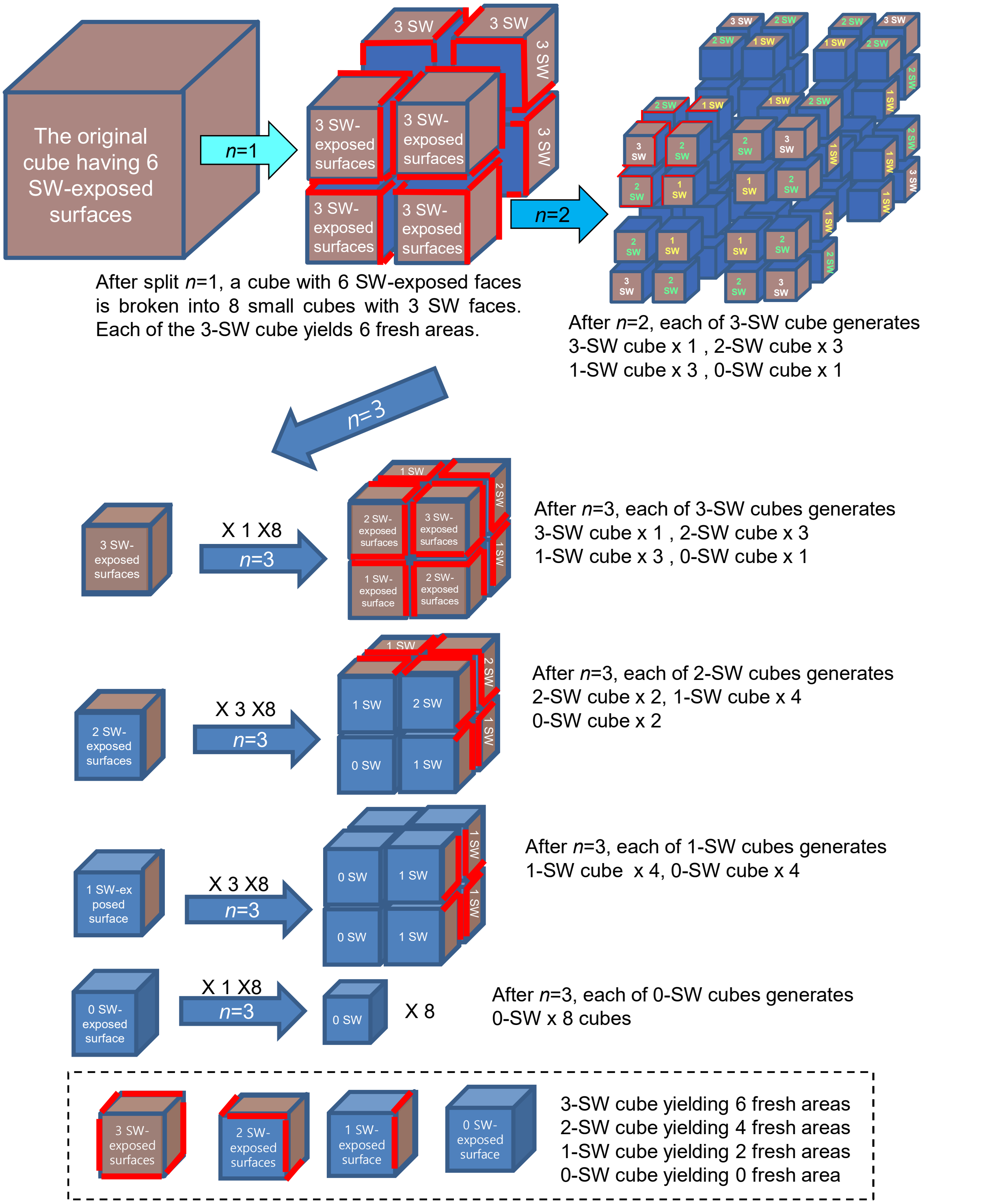

Fig. S2. Conceptual diagram of the calculation of the isotropic fragmentation of the Ryugu grains in the sample container. For example, cubes generated by fragmentation at $n = 1 – 3$ are shown. The red lines indicate the fresh surface areas (cross-sections of the SW-containing layers) generated by fragmentation. SW noble gases are released from these fresh areas.

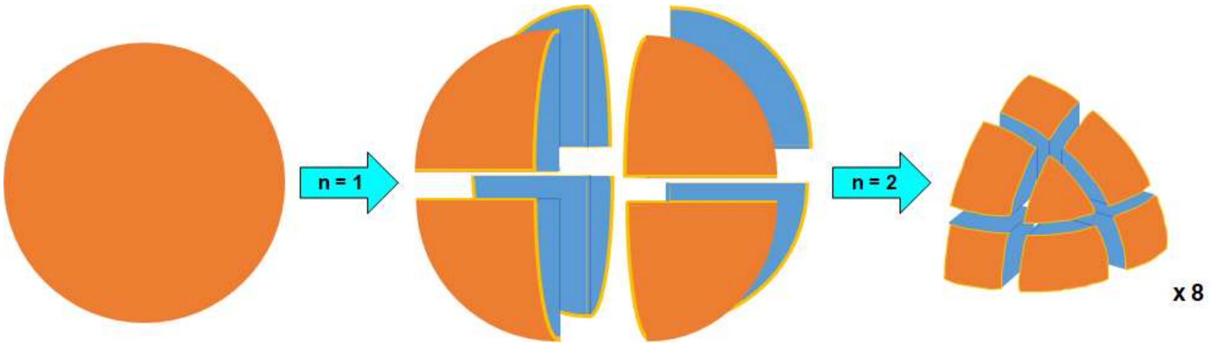

Fig. S3. Fragmentation model of spherical Ryugu grains. For $n > 1$, variety in the shape of the fragments occur.

Table. S1. Compositions of noble gases and volatile compounds in the Hayabusa2 sample container.

Pipette number	Type of pipet	^{4}He cm^{3}STP	$^{3}He/^{4}He$ ×10^{-4}	^{20}Ne cm^{3}STP	$^{20}Ne/^{22}Ne$	$^{21}Ne/^{22}Ne$	^{36}Ar cm^{3}STP	^{40}Ar cm^{3}STP	$^{38}Ar/^{36}Ar$	$^{40}Ar/^{36}Ar$	^{84}Kr cm^{3}STP	^{132}Xe cm^{3}STP
**CRPG-CNRS, Nancy**												
NT1P2E	Haya2	3.22E-05	1.510 ± 0.040	2.01E-06	10.398 ± 0.030	0.03027 ± 0.00053	3.33E-06	9.32E-04	0.1847 ± 0.0011	280.0 ± 2.5	3.18E-08	2.51E-09
NT1P2E	Haya2			2.66E-06	10.250 ± 0.050	0.03020 ± 0.00090	3.83E-06	1.09E-03	0.18380 ± 0.00070	284.68 ± 0.40		
NT1P2E	Haya2			3.10E-06	10.270 ± 0.050	0.02990 ± 0.00090	4.45E-06	1.28E-03	0.18420 ± 0.00070	284.30 ± 0.40		
NT5P2G	Blank	2.05E-08	n.d.	4.11E-09	n.d.	n.d.	n.d.	3.39E-07	n.d.	n.d.	1.19E-10	1.21E-11
**ETH Zürich**												
NT1P4Q1	Haya2	3.80E-05	1.458 ± 0.056	4.24E-06	10.312 ± 0.035	0.02948 ± 0.00039	6.65E-06	1.83E-03	0.1825 ± 0.0052	275.5 ± 2.1	8.94E-08	9.54E-09
NT1P5C	Haya2	2.62E-05	1.433 ± 0.086	2.96E-06	10.260 ± 0.065	0.02882 ± 0.00048	4.45E-06	1.24E-03	0.1820 ± 0.0025	278.2 ± 2.7	6.15E-08	6.52E-09
NT1P5D	Haya2	2.69E-05	1.421 ± 0.064	2.93E-06	10.208 ± 0.074	0.02920 ± 0.00042	4.44E-06	1.24E-03	0.1764 ± 0.0043	279.2 ± 2.6	6.17E-08	6.51E-09
NT5P3Q2	Blank	n.d.		1.62E-09	7.1 ± 6.4	n.d.	4.09E-08	1.22E-05	0.1876 ± 0.0021	297.8 ± 2.9	1.60E-10	1.79E-11
**Washington Univ.**												
NT1P3B	Haya2	2.88E-05		1.69E-06	10.340 ± 0.030	0.02962 ± 0.00020	3.03E-06	8.13E-04	0.1862 ± 0.0011	268.4 ± 1.0	3.64E-08	3.64E-09
NT1P3B	Haya2	2.73E-05		1.58E-06	10.344 ± 0.022	0.02978 ± 0.00011	2.87E-06	7.75E-04	0.1834 ± 0.0019	269.8 ± 1.5	3.30E-08	3.46E-09
NT1P3B	Haya2		1.36 ± 0.10									
**Kyushu Univ.**												
NT1P4C	Haya2	2.437E-05	2.10 ± 0.23	3.40E-06	10.393 ± 0.020	0.02992 ± 0.00044	4.22E-06	1.16E-03	0.1953 ± 0.0038	274 ± 10	6.21E-08	5.89E-09
NT5P3D	Blank	1.417E-09	9.3 ± 5.9	n.d.	n.d.	n.d.	4.7E-10	1.47E-07	0.204 ± 0.014	313 ± 40	n.d.	3.70E-13
**Averages**		2.91E-05	1.69 ±									

Pipette number	Type of pipet	^{84}Kr cm^{3}STP	$^{78}Kr/^{84}Kr$	$^{80}Kr/^{84}Kr$	$^{82}Kr/^{84}Kr$	$^{83}Kr/^{84}Kr$	$^{86}Kr/^{84}Kr$
**CRPG-CNRS, Nancy**							
NT1P2E	Haya2	3.18E-08	0.006340 ± 0.000080	0.04086 ± 0.00028	0.20530 ± 0.00077	0.20304 ± 0.00064	0.30309 ± 0.00098
NT5P2G	Blank	1.19E-10	n.d.	n.d.	n.d.	n.d.	n.d.
**ETH Zürich**							
NT1P4Q1	Haya2	8.94E-08	0.006355 ± 0.000079	0.04041 ± 0.00026	0.2048 ± 0.0010	0.20102 ± 0.00097	0.3022 ± 0.0015
NT1P5C	Haya2	6.15E-08	0.006376 ± 0.000076	0.04015 ± 0.00028	0.20362 ± 0.00087	0.20295 ± 0.00088	0.3012 ± 0.0013
NT1P5D	Haya2	6.17E-08	0.006499 ± 0.000085	0.04030 ± 0.00030	0.20388 ± 0.00096	0.20120 ± 0.00094	0.3002 ± 0.0015
NT5P3Q2	Blank	1.60E-10	n.d.	n.d.	n.d.	n.d.	n.d.
**Washington Univ.**							
NT1P3B	Haya2	3.64E-08	0.006675 ± 0.000059	0.04060 ± 0.00019	0.20432 ± 0.00074	0.20266 ± 0.00061	0.30325 ± 0.00058
NT1P3B	Haya2	3.30E-08	0.006612 ± 0.000062	0.04065 ± 0.00015	0.20392 ± 0.00042	0.20253 ± 0.00041	0.30307 ± 0.00057
**Kyushu Univ.**							
NT1P4C	Haya2	6.21E-08	0.00634 ± 0.00040	0.04170 ± 0.00082	0.2102 ± 0.0033	0.2070 ± 0.0028	0.3044 ± 0.0046
NT5P3D	Blank	n.d.	n.d.	n.d.	n.d.	n.d.	n.d.

Pipette number	Type of pipet	^{132}Xe cm^{3}STP	$^{124}Xe/^{132}Xe$	$^{126}Xe/^{132}Xe$	$^{128}Xe/^{132}Xe$	$^{129}Xe/^{132}Xe$	$^{130}Xe/^{132}Xe$	$^{131}Xe/^{132}Xe$	$^{134}Xe/^{132}Xe$	$^{136}Xe/^{132}Xe$
**CRPG-CNRS, Nancy**										
NT1P2E	Haya2	2.51E-09	0.00361 ± 0.00027	0.00338 ± 0.00030	0.0714 ± 0.0028	0.971 ± 0.014	0.1519 ± 0.0048	0.796 ± 0.012	0.3883 ± 0.0067	0.3297 ± 0.0064
NT5P2G	Blank	1.21E-11	n.d.	n.d.	n.d.	n.d.	n.d.	n.d.	n.d.	n.d.
**ETH Zürich**										
NT1P4Q1	Haya2	7.77E-09	0.00348 ± 0.00014	0.00362 ± 0.00015	0.07163 ± 0.00066	0.9862 ± 0.0066	0.1545 ± 0.0013	0.7932 ± 0.0060	0.3886 ± 0.0028	0.3320 ± 0.0024
NT1P5C	Haya2	6.52E-09	0.00325 ± 0.00016	0.00304 ± 0.00015	0.07199 ± 0.00078	0.9881 ± 0.0066	0.1534 ± 0.0014	0.7949 ± 0.0057	0.3858 ± 0.0031	0.3266 ± 0.0024
NT1P5D	Haya2	5.04E-09	0.00355 ± 0.00018	0.00340 ± 0.00013	0.0724 ± 0.0011	0.9868 ± 0.0072	0.1531 ± 0.0014	0.7887 ± 0.0051	0.3895 ± 0.0030	0.3259 ± 0.0027
NT5P4Q2	Blank	1.46E-11	n.d.	n.d.	0.089 ± 0.065	1.22 ± 0.31	0.187 ± 0.090	0.92 ± 0.26	0.39 ± 0.19	0.181 ± 0.082
**Washington Univ.**										
NT1P3B	Haya2	3.12E-09	0.003648 ± 0.000098	0.003321 ± 0.000056	0.07257 ± 0.00043	0.9888 ± 0.0030	0.15135 ± 0.0011	0.7895 ± 0.003	0.3885 ± 0.002	0.3294 ± 0.0028
NT1P3B	Haya2	2.97E-09	0.00347 ± 0.00013	0.003346 ± 0.000056	0.07126 ± 0.00045	0.9827 ± 0.0035	0.15168 ± 0.00093	0.7876 ± 0.0026	0.3890 ± 0.0015	0.3296 ± 0.0014
**Kyushu Univ.**										
NT1P4C	Haya2	4.79E-09	0.00308 ± 0.00025	0.00331 ± 0.00016	0.071 ± 0.0013	0.9970 ± 0.0070	0.1536 ± 0.0019	0.7944 ± 0.0051	0.3832 ± 0.0054	0.3230 ± 0.0052
NT5P3D	Blank	3.18E-13	n.d.	n.d.	n.d.	n.d.	n.d.	n.d.	n.d.	n.d.

Pipette number	Type of pipet	H_2 [nmol]	O_2 [nmol]	CH_4 [nmol]	$\delta^{13}C$ of CH_4	H_2O [nmol]	N_2 [μmol]	$\delta^{15}N$ of N_2	CO [nmol]	C_2H_6 [nmol]	CO_2 [nmol]
**Tokyo Inst. of Technology**											
NT1P1A	Haya2		n.d.	37 ± 16	n.d.	n.d.	7.06 ± 0.59	n.d.	n.d.	1.18 ± 0.3	n.d.
NT5P1C	Blank		n.d.	36 ± 29	n.d.	n.d.	n.d.	n.d.	n.d.	1.47 ± 0.3	n.d.
**JAMSTEC**											
NT1P1B	Haya2	10.62 ± 2.58		n.d.	-49.0 ± 3.2						
**Ibaraki U.**											
NT1P2F	Haya2						4.139	-14.4 ± 0.7			
NT1P2F	Haya2						4.269	-16.1 ± 0.7			
NT1P2F	Haya2						4.269	-14.2 ± 0.7			
NT1P2F	Haya2						4.367	-15.4 ± 0.7			
NT1P2F	Haya2						5.051	-16.6 ± 0.7			
Average of NT1P2F							4.432 ± 0.358	-15.3 ± 1.0			
NT5P2H	Blank						5E-04				
NT5P2H	Blank						9E-04				
NT5P2H	Blank						7E-04				
Average of NT5P2H							7E-04 ± 2E-04				
**CRPG-CNRS, Nancy**											
NT1P2E	Haya2						4.41 ± 0.15	-13.00 ± 0.50			
NT1P2E	Haya2						5.79 ± 0.19	-13.80 ± 0.50			
Average of NT1P2E							5.10 ± 0.69	-13.40 ± 0.57			
NT5P2G	Blank						0.008 ± 0.002	3.7 ± 5.1			
NT5P2G	Blank						0.010 ± 0.003	7.0 ± 5.1			
Average of NT5P2G							0.009 ± 0.001	5.4 ± 1.7			

	$^3He/^4He \times 10^{-4}$	$^{20}Ne/^{22}Ne$	$^{21}Ne/^{22}Ne$	$^{20}Ne/^4He$	$^{36}Ar/^4He$	$^{36}Ar/^{20}Ne$
**Average of Haya2 gas***	1.428 ± 0.010	10.3427 ± 0.0050	0.02980 ± 0.00015	0.1099 ± 0.0037	0.1661 ± 0.0048	1.5102 ± 0.0094

Errors of individual measurements are 1σ.

*The average isotopic ratios of the sample container gas were calculated by weighted averaging of the measurements from the different laboratories. Dispersions of $^3He/^4He$ ratios ($1/\sigma^2$) of individual measurements were used as the weighting factor for $^3He/^4He$ and elemental ratios, except for Ne isotopic ratios (where dispersions of $^{20}Ne/^{22}Ne$ and $^{21}Ne/^{22}Ne$ ratios were used).

Table. S2. Calculation of the fresh SW surface area generated by the isotropic fragmentation.

Fragment. factor *n*	No. of grains	Side length [cm]	No. of cubes generated				Fresh SW surface [cm^2]	Cumulative surface [cm^2]
			3-SW	2-SW	1-SW	0-SW		
START	1	1	-	-	-	-	-	-
1	8	0.5	1	0	0	0	1.20E-04	1.20E-04
2	64	0.25	1	3	3	1	2.40E-04	3.60E-04
3	512	0.125	1	9	27	27	4.80E-04	8.40E-04
4	4096	0.0625	1	21	147	343	9.60E-04	1.80E-03
5	32768	0.03125	1	45	675	3375	1.92E-03	3.72E-03

It is assumed that the original side length is 1 cm and the thickness of the solar wind (SW) concentrated layer is 50 nm. "3-SW" means a cube with 3 SW-exposed faces, and "2-SW" means a cube with 2 SW-exposed faces, and so on. "No. of cubes generated" in the 4th column means the number of cubes generated from each fragment, i.e., the total number of cubes is calculated by multiplying the figure of each cube type by 8 (e.g., for $n = 2$, the total cube number is $(1+3+3+1)\times 8 = 64$). "Fresh SW surface" is area generated at each fragmentation, while "Cumulative surface" is an integrated value of "Fresh SW surface" from which SW gases is expected to be released. Fragmentation should stop when the side length reaches 0.125 cm that is the present (actual) grain size of Ryugu samples (*10*).
